# Histopathological characterization of inflammatory interparietal involvement in frontal fibrosing alopecia

**DOI:** 10.3389/fmed.2025.1740090

**Published:** 2026-01-12

**Authors:** Marta Gonzalez Cañete, Ana Rodríguez-Villa Lario, David Vega Díez, María Dolores Vélez Velazquez, Jose Maria Ricart Vaya, Alba Gómez Zubiaur

**Affiliations:** 1 Instituto médico ricart, Madrid, Spain; 2 Department of Dermatology, Hospital Universitario Principe de Asturias, Alcala de Henares, Spain; 3 Department of Pathology, Hospital Universitario Principe de Asturias, Alcala de Henares, Spain

**Keywords:** AGA, androgenetic alopecia, CA-FFA, FAPD, FFA, FFA with crown-area involvement, fibrosing alopecia with a pattern distribution, frontal fibrosing alopecia

## Abstract

**Introduction:**

Frontal fibrosing alopecia (FFA) may involve the interparietal area; however, histopathologic features in this site remain undefined.

**Objective:**

The objective of the study was to histopathologically characterize inflammatory interparietal involvement in patients with FFA in real-world practice.

**Methods:**

A descriptive, retrospective study enrolled patients with FFA who developed interparietal inflammatory findings. Patients underwent two interparietal biopsies; samples were assessed under a uniform protocol, which included follicular counts, inflammatory infiltrate features, scar tissue, vacuolar degeneration (VD) of the follicular epithelium, epithelial or stromal (E/S) clefting, concentric lamellar fibroplasia (CLF), and sebaceous glands (SGs).

**Results:**

A total of 12 females (mean age: 64.25 years) were included. Following dermatopathologic review, six patients were classified as having crown-area FFA (CA-FFA), four patients as having fibrosing alopecia with a pattern distribution (FAPD), and two patients as having indeterminate cicatricial alopecia. CA-FFA showed greater structural loss with scar tissue and sebaceous gland reduction, whereas FAPD showed a miniaturization-dominant profile and increased catagen/telogen hairs. Patients with a biopsy suggestive of CA-FFA more often had a diffuse clinical pattern, were older, had a longer disease duration, and had approximately twice as much occipital involvement than as those in the FAPD group.

**Discussion/Conclusion:**

Given that FFA produces greater structural disruption than FAPD, our results raise the question of whether these conditions are overlapping entities or part of the same process that has progressed. Further studies in patients with FFA and FAPD are needed to elucidate the role of androgenetic factors in addition to lymphohistiocytic infiltrate and perifollicular lamellar fibrosis.

## 1 Introduction

Frontal fibrosing alopecia (FFA) is an acquired lymphocytic scarring alopecia of increasing incidence worldwide. It is clinically characterized by a progressive frontotemporal hairline recession, often accompanied by eyebrow alopecia. Occasionally, FFA may present with interparietal involvement, a feature that Kossard already reported in 1994 (1). This involvement may reflect coexistence of FFA with androgenetic alopecia (AGA) (2), fibrosing alopecia with a pattern distribution (FAPD) (3), or lichen planopilaris (LPP) (2), or it may be due to the disease itself (4–6), which we refer to as FFA with crown-area involvement (CA-FFA). Trichoscopic evaluations of patients with FFA and interparietal involvement have identified a diffuse fibrotic pattern in up to 68% of patients (6). However, in the absence of histopathologic confirmation, it remains unclear how many cases truly correspond to CA-FFA or represent an overlap with other alopecia types. To the best of our knowledge, no previous histopathological studies have specifically addressed interparietal involvement in FFA. Clarifying these histopathologic patterns may assist in distinguishing CA-FFA from overlapping forms of scarring alopecia, improving diagnostic accuracy.

## 2 Materials and methods

We conducted a descriptive and retrospective study enrolling patients with a previous clinical and trichoscopic diagnosis of FFA, in whom interparietal inflammatory findings were observed during follow-up. Participants were evaluated at the Trichology Unit of our center between July and September 2021.

Inflammatory findings were defined as clinical and/or trichoscopic evidence of interparietal involvement and/or symptomatology in that area, including reduced hair density, pruritus, or signs of perifollicular inflammation or scarring, such as perifollicular erythema, perifollicular hyperkeratosis, or loss of follicular ostia.

Two 4-mm interparietal punch biopsies guided by trichoscopy were performed in all patients. The specimens were fixed in formaldehyde, processed in vertical and horizontal sections, and stained with hematoxylin and eosin. The samples were analyzed by the same expert dermatopathologist, following the same protocol. The number of terminal follicles (TFs), miniaturized follicles (MFs), the presence and characteristics of the inflammatory infiltrate, scar tissue, the presence or absence of pigmentary molds, vacuolar degeneration of the follicular epithelium, epithelial or stromal clefting, concentric lamellar fibroplasia, and sebaceous glands were studied in all samples. Informed written consent was obtained from all patients.

This study aimed to characterize the histopathology in the interparietal region of patients with FFA and clinical and/or trichoscopic findings suggestive of inflammation in the interparietal region and/or symptoms in the said location in real clinical practice.

## 3 Results

A total of 12 Caucasian females with a previous diagnosis of FFA and with a mean age of 64.25 years (range: 49–83) were included. Comorbid conditions included hypothyroidism (*n* = 5), cutaneous lichen planus (*n* = 1), breast cancer (*n* = 1), rosacea (*n* = 1), psoriasis (*n* = 1), and genital lichen sclerosus (*n* = 1).

**Figure 1:**
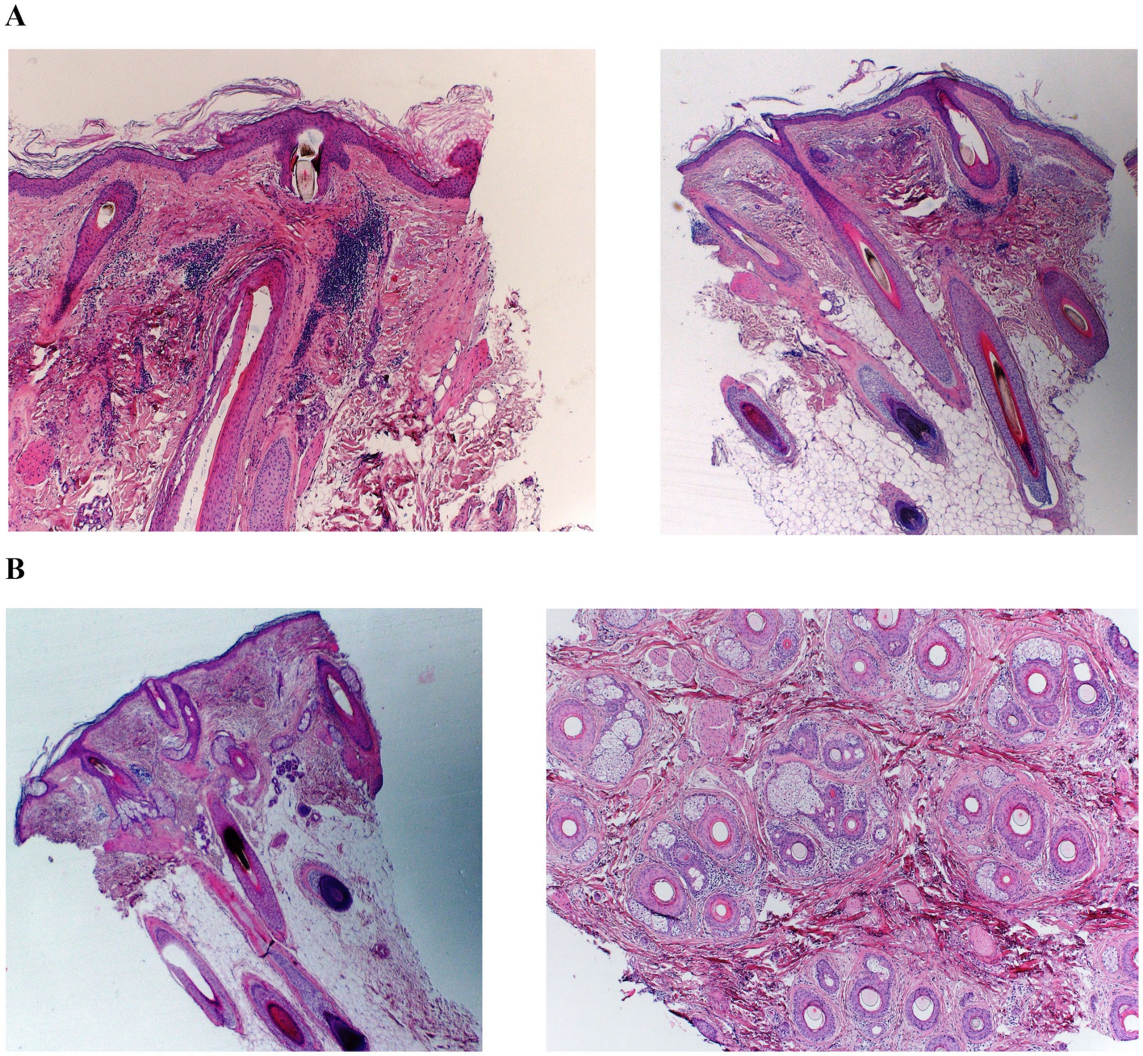
Hematoxylin–eosin (H&E), transverse and horizontal sections. **(A)** CA-FFA (patient 1): terminal anagen follicles with absence of vellus follicles and absent sebaceous glands, associated with scar tissue and a superficial peri-infundibular lymphocytic infiltrate. **(B)** FAPD (patient 11): both terminal and vellus/miniaturized follicles, some with superficial lymphocytic infiltrates, together with concentric lamellar fibroplasia.

Following blinded dermatopathologic review, six patients were classified as having findings consistent with CA-FFA (Figure 1A), four patients as having FAPD (Figure 1B), and two patients could not be assigned to either group; however, they both had a cicatricial alopecia with lymphocytic infiltrates. Patients were classified as FAPD if biopsies showed an increased number of vellus hairs, an increase in terminal follicles in catagen and telogen, and a lower terminal-to-vellus ratio (7), and they were classified as CA-FFA if biopsies showed a higher number of follicular scars, more vacuolar degeneration of the follicular epithelium, a greater presence of follicular clefts, and fewer arrector pili muscles (7).

Detailed histopathologic findings are shown in Table 1. Scar tissue and concentric lamellar fibroplasia were observed consistently across the groups. The inflammatory infiltrate was variable and generally mild to moderate, most often superficial (peri)infundibular, and was absent in some samples. Vacuolar degeneration of the follicular epithelium was uncommon (observed in a single CA-FFA case), and epithelial–stromal clefting occurred infrequently, exclusively within CA-FFA. Sebaceous glands were typically absent or deeply reduced in CA-FFA, reduced but present in FAPD, and focally reduced/normal in indeterminate cases. FAPD biopsies contained more vellus/miniaturized hairs with a shift of terminal follicles toward catagen/telogen, whereas CA-FFA showed greater structural loss.

**Table 1:** Histopathologic findings in interparietal biopsies.

Patient	Total follicles	TF	V/M F	TF anagen	TF telogen	TF catagen	Inflammatory infiltrate	Scar tissue	PM	VD	E/S C	CLF	SG	Dx
1	13	13	0	12	1	0	Infund. mod.	+	−	−	+	+	−	CA-FFA
2	14	12	2	14	0	0	None	+	−	−	−	+	↓↓	CA-FFA
3	21	17	4	19	0	2	M/VF, mild	+	−	−	+	+	↓	FAPD
4	27	22	5	25	1	1	TF and M/VF peri-infund. Mild–mod.	+	−	−	−	+	↓	FAPD
5	19	14	3	18	0	1	Perifollicular sup. mild	+	−	−	−	+	↓	NE^1^
6	28	6	22	20	5	3	None	+	−	−	−	+	↓	FAPD
7	34	29	5	34	0	0	TF and M/VF peri-infund. mild	+	−	−	+	+	↓	CA-FFA
8	23	21	2	22	0	1	Peri-infund. mild	+	−	−	−	+	−	CA-FFA
9	22	19	3	19	0	0	None	+	−	−	−	+	−	CA-FFA
10	18	18	0	17	0	1	Sup. mod.	+	−	+	+	+	−	CA-FFA
11	34	22	12	32	0	2	M/VF sup. mild	+	−	−	−	+	↓	FAPD
12	26	24	2	21	0	5	Perivascular infiltrate/sup.	+	−	−	−	+	↓ (focal)	NE^2^

TF, terminal follicles; V/MF, vellus or miniaturized follicles; PM, pigmented molds; VD, vacuolar degeneration of the follicular epithelium; E/S C, epithelial or stromal clefting, CLF, concentric lamellar fibroplasia; SG, sebaceous glands; Dx, diagnosis; +/−, present/absent; sup., superficial; mod., moderate; ↓, reduced; ↓↓, markedly reduced. NE^1^: cicatricial alopecia with terminal follicles and some vellus follicles, preservation of sebaceous glands, and mild superficial perifollicular lymphocytic infiltrates. NE^2^: cicatricial alopecia with increased catagen follicles, concentric lamellar fibroplasia in some terminal follicles, and mild perifollicular lymphocytic infiltrate.

Patients classified as CA-FFA were older (mean age: 70 vs. 57 years in FAPD) and had longer median disease duration (9 vs. 3 years). Clinical patterns of FFA in the study population were as follows: in the CA-FFA group (*n* = 6), two patients exhibited Type 1 patterns and four patients exhibited Type 2 patterns; in the FAPD group (*n* = 4), two patients exhibited Type 1 patterns, one patient exhibited Type 2 patterns, and one patient exhibited Type 3 patterns; and in the indeterminate group (*n* = 2), one patient exhibited Type 1 patterns and one patient exhibited Type 2 patterns. Overall, diffuse or Type 2 patterns were observed in 66.7% of CA-FFA patients, compared with only 25% of FAPD patients.

Occipital involvement was present in 50% of CA-FFA patients and 25% of FAPD patients; all patients had eyebrow alopecia, and 50% in both groups had body hair involvement. Preauricular wrinkling was the most frequent accompanying facial manifestation across all groups. The majority of participants exhibited a diffuse pattern of clinical interparietal involvement, with the exception of one patient classified as CA-FFA, whose case prompted a reconsideration of the diagnosis, since she exhibited macroscopically visible alopecia plaques, raising the possibility of an overlap between FFA and LPP. Interparietal pruritus was reported by 83% of CA-FFA patients and 50% of FAPD patients. Demographic and clinical characteristics are shown in Table 2. In terms of trichoscopy, the majority of patients in the FAPD group showed evidence of hair miniaturization. The most frequent finding across all groups was perifollicular desquamation.

**Table 2:** Demographic and clinical characteristics (per patient).

Patient	Age (years)	Disease duration (years)	Comorbidities	Clinical pattern (Type 1/2/3)	Eyebrow alopecia	Occipital involvement	Body hair involvement	Interparietal involvement type	Interparietal pruritus	Accompanying features^*^	Diagnosis
1	73	6	Hypothyroidism	Type 1	Yes	Yes	Yes	Diffuse	No	Preauricular wrinkling	CA-FFA
2	83	12	Hypothyroidism	Type 2	Yes	No	Yes	Plaques	Yes	Preauricular wrinkling and frontal vein prominence	CA-FFA
3	54	2	Cutaneous lichen planus	Type 3	Yes	No	No	Diffuse	Yes	Preauricular wrinkling and frontal vein prominence	FAPD
4	49	3	–	Type 1	Yes	No	Yes	None	Yes	Facial papules	FAPD
5	57	10	–	Type 1	Yes	Yes	No	Diffuse	Yes	Facial papules	Indeterminate
6	62	10	Hypothyroidism and genital lichen sclerosus	Type 1	Yes	Yes	Yes	Diffuse	No	Preauricular wrinkling	FAPD
7	56	8	–	Type 2	Yes	No	No	Diffuse	Yes	Preauricular wrinkling and frontal vein prominence	CA-FFA
8	62	7	Breast cancer	Type 1	Yes	No	No	Diffuse	Yes	Preauricular wrinkling and extrafacial follicular red dots	CA-FFA
9	69	10	Rosacea	Type 2	Yes	Yes	No	Diffuse	Yes	Frontal vein prominence	CA-FFA
10	77	30	Psoriasis and hypothyroidism	Type 2	Yes	Yes	Yes	Diffuse	Yes	–	CA-FFA
11	63	3	Hypothyroidism	Type 2	Yes	No	Yes	Diffuse	No	–	FAPD
12	66	1	–	Type 2	Yes	Yes	No	Diffuse	Yes	–	Indeterminate

^*^Accompanying features grouped as present on clinical examination: facial papules, preauricular wrinkling, extrafacial follicular red dots, and frontal vein prominence.

All patients underwent treatment prior to biopsy (Table 3). All but one patient were on systemic therapy, with the vast majority receiving dutasteride (either as monotherapy or in combination). Additionally, seven patients were treated topically in the interparietal region with corticosteroids and/or 5% minoxidil. Treatment distribution was generally balanced across the study groups.

**Table 3:** Treatments at the time of biopsy.

Patient	Interparietal topical treatment	Frontal topical treatment	Systemic treatment
1	Clobetasol, minoxidil, and finasteride	Clobetasol, minoxidil, and finasteride	Dutasteride and isotretinoin
2	Mometasone furoate	Mometasone furoate	
3		Pimecrolimus	Dutasteride and minoxidil
4	Minoxidil	Pimecrolimus	Dutasteride
5	Mometasone furoate	Pimecrolimus and mometasone furoate	Dutasteride
6	Clobetasol	Pimecrolimus and clobetasol	Dutasteride and azithromycin
7		Pimecrolimus	Dutasteride and isotretinoin
8		Pimecrolimus	Minoxidil and isotretinoin
9	Minoxidil and finasteride	Pimecrolimus, minoxidil, and finasteride	Dutasteride
10	Clobetasol and minoxidil	Pimecrolimus, clobetasol, and minoxidil	Dutasteride and isotretinoin
11			Dutasteride and minoxidil
12	Clobetasol	Pimecrolimus and clobetasol	Dutasteride

## 4 Discussion

After examining interparietal scalp biopsies from patients with a prior diagnosis of FFA who, on follow-up, developed clinical or trichoscopic evidence of inflammatory involvement in that region, we identified two main histopathologic patterns: CA-FFA and FAPD. CA-FFA was characterized by greater structural loss with reduced/absent sebaceous glands and occasional epithelial–stromal clefting. In contrast, FAPD showed more vellus/miniaturized hairs and a shift of terminal follicles toward catagen/telogen, with comparatively milder architectural disruption. Patients with a biopsy suggestive of CA-FFA more often had a diffuse clinical pattern, which implies a worse prognosis, were older, had a longer disease duration, and had approximately twice as much occipital involvement as than those in the FAPD group.

Our results, together with prior observations that FFA causes a greater structural disruption than FAPD (7), raise the question of whether FFA and FAPD represent overlapping entities, as already proposed by other authors, or whether they are part of the same process that has progressed (8, 9). Familial occurrence of both FAPD and FFA has been reported in the literature (10), and the presence in FAPD of features otherwise associated with FFA—such as facial papules (11) and/or extrafacial follicular red dots (12)—prompts us to consider if there is a common pathogenic pathway between both entities.

Furthermore, different authors have proposed considering both entities within a single spectrum. Du et al. described five patients with focal and diffuse fibrosing alopecia with overlapping features and a slowly progressive course (8). However, in contrast to the findings of our study, they noted that younger patients had more evident thinning at the hairline and comparatively mild involvement at the crown, whereas older patients exhibited advanced thinning at both locations. Other authors have proposed that FFA and FAPD may represent the end of a continuum starting from AGA with histologic evidence of follicular microinflammation culminating in clinically noticeable forms of inflammatory cicatricial alopecia (9). They have reported that this hypothesis is reinforced by the overlap of FFA, AGA, and FAPD observed in 11% of their patients, a hypothesis that would also be reinforced by our results.

Moreover, one study found no statistically significant difference in the presence of perifollicular inflammatory infiltrates in affected and unaffected areas of the scalp in patients with FFA (13), while another study showed infundibular and isthmic perifollicular infiltrates in 88% of patients with AGA (14). The reason why these infiltrates do not produce scarring alopecia is still unknown. A plausible explanation is that an as-yet unidentified antigenic stimulus from the damaged hair follicle may trigger a lichenoid tissue reaction in immunologically susceptible individuals (9).

Our study has some limitations. First, the sample size was small, and we did not have a control group. Second, we did not characterize the non-inflammatory interparietal involvement of patients with FFA. Third, our patients underwent treatment prior to the biopsy, which might alter the histopathological results. We may have observed lower-dense inflammatory infiltrates due to the use of corticosteroids and other anti-inflammatory drugs, an increase in anagen follicles due to minoxidil, and a decrease in miniaturized hairs due to the use of 5-alpha-reductase inhibitors. These factors should be considered when interpreting our findings.

## 5 Conclusion

To the best of our knowledge, we present the first study whose objective is to histopathologically characterize inflammatory interparietal involvement in patients with FFA, having found mainly two patterns. Patients with CA-FFA more frequently presented a diffuse clinical pattern, were older, and had a higher median evolution time than those belonging to the FAPD group.

Further studies in patients with FFA and FAPD are needed to elucidate the presumed role of androgenetic factors in addition to the lymphohistiocytic infiltrate and perifollicular lamellar fibrosis. It remains to be established whether differentiating between FFA and FAPD in the interparietal area may have prognostic and therefore therapeutic implications.

## Data Availability

The raw data supporting the conclusions of this article will be made available by the authors, without undue reservation.
